# Direct measurement of strain-dependent solid surface stress

**DOI:** 10.1038/s41467-017-00636-y

**Published:** 2017-09-15

**Authors:** Qin Xu, Katharine E. Jensen, Rostislav Boltyanskiy, Raphaël Sarfati, Robert W. Style, Eric R. Dufresne

**Affiliations:** 1 Department of Materials, ETH Zürich, 8093 Zürich, Switzerland; 2 Department of Mechanical Engineering and Materials Science, Yale University, New Haven, CT 06511 USA; 3 Mathematical Institute, University of Oxford, Oxford, OX1 3LB UK

## Abstract

Surface stress, also known as surface tension, is a fundamental material property of any interface. However, measurements of solid surface stress in traditional engineering materials, such as metals and oxides, have proven to be very challenging. Consequently, our understanding relies heavily on untested theories, especially regarding the strain dependence of this property. Here, we take advantage of the high compliance and large elastic deformability of a soft polymer gel to directly measure solid surface stress as a function of strain. As anticipated by theoretical work for metals, we find that the surface stress depends on the strain via a surface modulus. Remarkably, the surface modulus of our soft gels is many times larger than the zero-strain surface tension. This suggests that surface stresses can play a dominant role in solid mechanics at larger length scales than previously anticipated.

## Abstract (web summary)

Solid surface stress is a fundamental property of solid interfaces. Here authors measure the solid surface stress of a gel, and show its dependence on surface strain through a surface modulus.

## Introduction

All material surfaces are characterized by a surface energy and a surface stress. The difference between these material properties is important: the surface energy, *γ*, is a scalar equal to the minimum work per unit area to cut a solid, whereas the surface stress, ϒ_*ij*_, is a tensor that describes the in-plane force per unit length required to stretch a surface. In simple liquids, the surface stress and surface energy have the same magnitude (ϒ_*ij*_ = *γδ*_*ij*_) and are independent of any deformation. In solids, both quantities are expected to be strain dependent. For small deformations, they are related through the Shuttleworth equation^1^,

1 $${\Upsilon _{ij}} = \gamma {\delta _{ij}} + \frac{{\partial \gamma }}{{\partial \epsilon _{ij}^s}},$$

where *δ*_*ij*_ is the identity tensor and $\epsilon _{ij}^s$ is the surface strain tensor. For nearly 60 years, Eq. (1) has served as the foundation for a well-established body of theory and computation including rigorous analyses of how surface stress can be incorporated into physical models^2, 3^, predictions for ${\Upsilon _{ij}}( {\epsilon _{ij}^s} )$ in a variety of materials^4–6^, and an extensive literature anticipating the role of strain-dependent surface stresses across various phenomena in nanostructures and nanocomposites^7–12^. Apart from some indications that ϒ_*ij*_ *≠* *γδ*_*ij*_ (e.g., see refs. ^13, 14^), there is sparse direct experimental evidence to support these theories. In particular, we are unaware of any experiments that have been able to measure strain-dependent surface stresses in a solid material^15^.

Here we directly measure surface stress as a function of strain in a compliant polymer gel. In such materials, surface stresses can be important at the micrometer scale and can be directly measured from the structure of a three-phase contact line using a light microscope^16^. In the range of accessible strains (up to 25%), surface stresses increase linearly. The effect is not subtle: surface stress doubles with only 17% strain.

## Results

### Structure of a soft solid contact

**Fig. 1:**
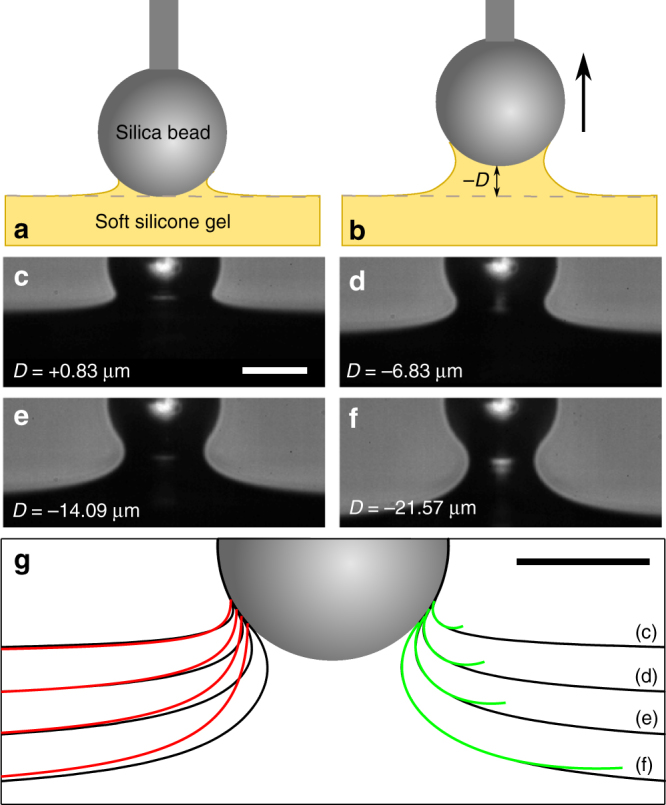
A solid meniscus in soft adhesion. **a**, **b** Schematic of the experiment at **a** initial contact and **b** during quasi-static pull. **c**–**f** Raw snapshots of a 17.4-μm-radius sphere adhered to an initially flat, compliant (*E* = 5.6 kPa) silicone gel substrate as it is pulled quasi-statically from **c** first contact to **f** the last measured stable position (*scale bar* in **c**: 20 μm). **g** Mapped deformation profiles (*black points*) corresponding to (**c**)–(**f**), with predictions of classic elastic theory^17, 18^ overlaid at left (*red lines*), and best-fit constant total curvature surfaces overlaid at right (*green lines*) (*scale bar*: 20 μm)

To clearly visualize the impact of strain-dependent surface stress, we first image extreme deformations of a soft solid. We bring rigid glass spheres with radii from 7.9 to 32.1 μm into adhesive contact with ~300 μm-thick, compliant, silicone gel substrates (Young’s modulus *E* = 5.6 kPa) and quasi-statically retract them, as shown schematically in Fig. 1a, b. Below a critical displacement, a solid, axisymmetric bridge of silicone stably connects the spheres to the substrates, as shown in the example optical micrographs of Fig. 1c-f.

As we pull on such an adhesive contact, the solid silicone bridge between substrate and sphere takes on a remarkable shape, resembling a liquid meniscus. This not only differs from the predictions of classic linear elastic theory^17, 18^, shown as red lines at left in Fig. 1g, but also from recent large-deformation simulations of Neo-Hookean solids^19^. Alternatively, we test the correspondence with a liquid meniscus by fitting the profiles to surfaces of constant total curvature, shown as green curves at right in Fig. 1g. The curves capture the shape of the solid free surface near the contact line. In this example, at first contact (Fig. 1c) the total curvature is −0.29 μm^−1^. As the bead is retracted, the magnitude of the curvature drops, decreasing to −0.01 μm^−1^ at the last recorded stable position (Fig. 1f). It is noteworthy that, as the size of the domain of constant curvature increases, the curvature drops. Thus, the adhesion profile increasingly resembles adhesion to an infinite liquid substrate.

Why should the free surface assume a liquid-like shape near the contact line? Recent theory and experiment, reviewed in ref. ^20^, have found that surface stresses dominate bulk elastic stresses at wavelengths smaller than a characteristic elastocapillary length scale, ϒ/*E* ^20^. Thus, we expect a capillary-dominated near field within this distance of the three-phase contact line. The size of the domain of constant curvature at initial contact in Fig. 1c is 5.1 μm, defined as the path length over which the capillary solution fits the measured profile. Indeed, this is comparable to the previously measured elastocapillary length ϒ/*E* for this material, about 4 μm^21^. However, the size of the constant curvature domain does not remain constant with deformation. Rather, it increases dramatically with sphere displacement, reaching 31.8 μm at the last stable position in Fig. 1f.

The growth of this solid meniscus with increasing strain suggests a proportional increase in the elastocapillary length, ϒ/*E*. This can only be due to an increase in the surface stress with tensile strain, as Young’s modulus is constant up to ~10% strain and then increases slightly thereafter (see Supplementary Information). This suggests a more than sixfold increase in ϒ during the deformation in Fig. 1. However, this experimental geometry is not well-suited to a quantitative measurement of the relationship between surface stress and strain. The strain in the solid meniscus is highly inhomogeneous and the size of the domain of constant curvature depends on both the bulk and surface mechanical properties.

### Microscopic wetting profiles

**Fig. 2:**
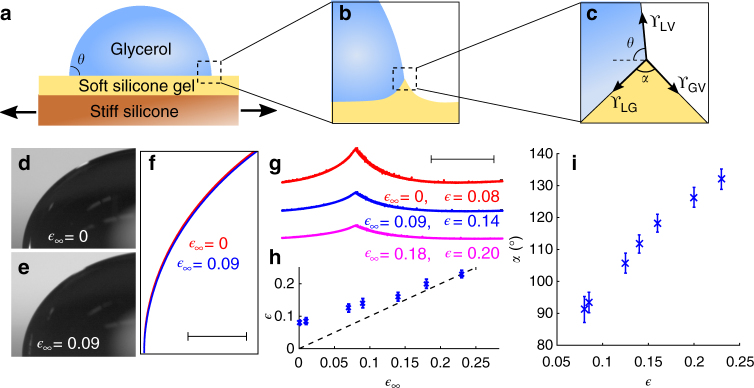
Macroscopic contact angle and microscopic wetting profiles. **a** Schematic of the strain-dependent wetting experiments, using a biaxial stretcher as described in ref. ^50^. **b** Detail of the contact line geometry at intermediate scales. **c** Detail of the contact line at microscopic scales, much less than ϒ/*E*. At this scale, the geometry of the contact line is given by a vector balance of the surface stresses as shown. **d**, **e** Macroscopic wetting profiles of large glycerol droplets sitting on unstretched and stretched (${\epsilon _\infty }$ = 0.09) silicone gels. **f** Superimposed boundaries for the drops on the stretched (*blue*) and unstretched (*red*) substrates show no difference in the macroscopic contact line geometry (*scale bar*: 400 μm). **g** Microscopic wetting profiles for a single droplet on unstretched (*red*), 9% stretched (*blue*), and 18% stretched (*pink*) silicone gel substrates, respectively (*scale bar*: 20 μm). **h** Local strain near the contact point, $\epsilon$, plotted against the applied strain, ${\epsilon _\infty }$. *Dashed line* has a slope of 1. **i** The opening angle of the wetting ridge, *α*, increases with the local strain, $\epsilon$. In **h**, **i**, the *error bars* are SD of the population

Instead, we designed wetting experiments that allow us to measure directly the local relationship between surface stress and strain. We measure the macroscopic and microscopic structure of the contact line of glycerol droplets on soft silicone substrates (*E* = 3.0 kPa) as we apply a uniform biaxial strain, ${\epsilon _\infty }$, as shown schematically in Fig. 2a–c. Although the macroscopic contact angle for large droplets depends only on the surface energies (Fig. 2a)^22^, the microscopic structure at the contact line with such a soft substrate is governed by a balance of surface stresses (Fig. 2c)^16, 23–25^.

Macroscopic measurements on large droplets show no change of the contact angle with applied strain; it always re-equilibrates to its original value of *θ* = 90.8° following a stretch (Fig. 2d–f and Supplementary Fig. 3). This demonstrates that there is no significant contact-line hysteresis, and that the surface energies of the solid–liquid and solid–vapor interfaces are nearly identical.

The microscopic structure of the contact line, on the other hand, reveals the surface stresses^16^. Within distances from the contact line of ϒ/*E*, each of the three intersecting interfaces becomes straight and has an orientation given by the mechanical balance of the surface stresses, as shown in Fig. 2c. Importantly, this shape is defined locally and is independent of the bulk elastic properties of the material. We measure the profile of the silicone substrate near the contact line using confocal microscopy, initially with zero applied strain. The substrate forms a symmetric wetting ridge at the contact line, as seen in Fig. 2g. With no applied strain, the ridge is ~10 μm high and has an opening angle *α* = 91.2°, determined by fitting the region close to the contact line as intersecting lines.

Knowing the shape of the substrate near the contact line, we can measure the local surface stress by applying the force balance of Fig. 2c. As the macroscopic contact angle is nearly 90°, we know that the liquid–air interface divides the opening angle *α* nearly equally and the horizontal force balance reduces to ϒ_LG_ ≈ ϒ_GV_ = ϒ^26^. Balancing the surface stresses along the vertical axis further requires that ϒ = ϒ_LV_/(2 cos(*α*/2)), where ϒ_LV_ is the liquid–vapor surface tension, which we measured to be 41 ± 1 mN m^−1^ for glycerol droplets in contact with the silicone substrate (see Methods section). The result is a measured solid surface stress of ϒ = 29 mN m^−1^, ~50% larger than the surface tension of silicone liquids and consistent with our previous measurements using a similar experimental geometry^16^.

In marked contrast to the macroscopic measurements, the microscopic contact line geometry changes significantly when we stretch the substrate. The contact line geometry for the same droplet at different values of applied biaxial strain, ${\epsilon _\infty }$, is shown in Fig. 2g. To ensure that the contact line has reached equilibrium, we wait at least 40 min after applying each strain. As the applied strain increases from 0 to 18%, the ridge height decreases by a factor of three and the opening angle increases to *α* = 126.3°. This change in the opening angle indicates that the surface stress has increased to 44 mN m^−1^, an ~50% increase from the value at ${\epsilon _\infty }$ = 0.

It is important to note that the strain at the contact line is a combination of the macroscopically applied strain, ${\epsilon _\infty }$, and the localized deformation that produces the wetting ridge. To meaningfully interpret the change in surface stress with applied strain, we measured the strain at the contact line, $\epsilon$, as a function of the applied strain, ${\epsilon _\infty }$ (as described in the Methods section). Even when the applied strain is zero (${\epsilon _\infty }$ = 0), the substrate is stretched in the wetting ridge with a local value of $\epsilon$ = 8% at the contact line. With increasing stretch, the wetting ridge flattens out and $\epsilon$ converges toward ${\epsilon _\infty }$, as shown in Fig. 2h.

Armed with the ability to extract local measures of both the surface stress and surface strain, we measured the wetting ridge profiles of 35 droplets having radii from 12 to 220 μm at seven different externally applied strains from 0 to 23% (Supplementary Fig. 4). As expected, the capillary-dominated structure near the contact line was identical for all droplets under the same strain conditions, even though the far-field profiles were elasticity dominated and depended on the droplet size. We plot the measured opening angle, *α*, vs. strain, $\epsilon$, for all experiments in Fig. 2i. As shown in Supplementary Fig. 5, the size of the capillary-dominated domain increases with applied strain, mirroring our observations from the adhesion experiment in Fig. 1g.

### Strain-dependent surface stress

**Fig. 3:**
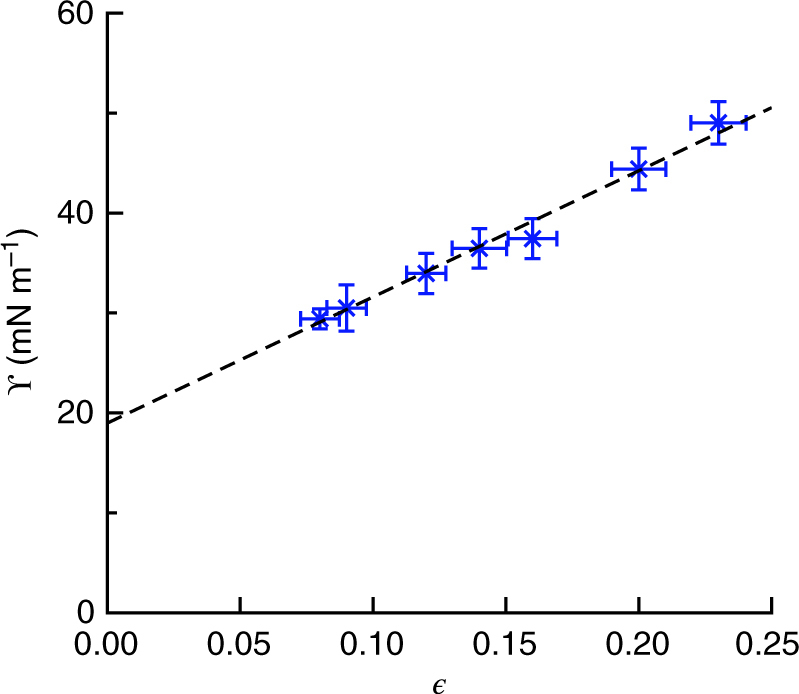
Strain dependence of solid surface stress, ϒ($\epsilon$). The *points* indicate the average surface stress and average local strain for droplets on the same substrate. The *error bars* are the SD of the population. The *dashed line* is a linear fit, providing the surface modulus, Λ = 126 ± 6 mN m^−1^ and zero-strain surface stress, ϒ_0_ = 19 ± 3 mN m^−1^

Applying the force balance of Fig. 2c to the data in Fig. 2i, we calculate the strain dependence of the solid surface stress, shown in Fig. 3. Over the range of measured strains, up to ~25%, we find that the surface stress increases linearly with strain. Fitting these data to the form ϒ = ϒ_0_ + Λ$\epsilon$, we find ϒ_0_ = 19 ± 3 mN m^−1^ and Λ = 126±6 mN m^−1^. The fitted value of the surface stress at zero strain, ϒ_0_, is quite close to the surface tension of the gel’s liquid silicone precursor, which we measured to be 21 mN m^−1^ (see Methods section).

The surface modulus, Λ, is a material property of the solid silicone gel surface. Surface moduli are predicted to have an important role in the nanoscale mechanics of metallic surfaces, but have never been measured directly^10, 11, 27^. As with bulk elasticity, elastic surfaces can be characterized by two surface moduli that represent the response of the surface to shear or biaxial stretch: the surface equivalents of shear and bulk moduli^20^. In our experiments, the strain is nearly isotropic, so Λ is solely dependent on the latter.

## Discussion

The strain dependence of the surface stress is remarkably strong, with $\Lambda \gg {\Upsilon _0}$. Consequently, the surface stress increases 2.5× with 25% strain. This dramatic effect emphasizes just how important strain-dependent solid surface stress is to a complete description of the mechanics of compliant materials. It may also resolve a persistent mystery in the reported values of the surface stress of soft silicones, which have thus far varied from 19 to 70 mN m^−1^, as each measurement has involved a different specific experimental geometry and thus different strain states (see Table 1).

**Table 1:** Measured values of surface stress of similar silicone gels published in the literature

**Silicone**	**Young’s Modulus (kPa)**	**Measured ϒ (mN** **m** ^**−1**^ **)**	**Reference**
Sylgard 184	770	19	^52^
Gelest	5.6	20	^21^
Sylgard 184	2400	26	^53^
Dow Corning CY52-276A/B	3	30	^16^
Sylgard 184	18	30–70	^28^
Sylgard 184	1000	40–50	^29^
Dow Corning CY52-276A/B	3	42–59	^23^

Decades of theoretical work have been based on the expectation that strain-dependent solid surface stress is a universal feature of all solids, but direct measurements in conventional stiff materials have proven extremely challenging. Soft solids, with their high compliance and ability to sustain large-strain elastic deformation, provide unique experimental model systems that make accessible direct measurements of this fundamental material property. Moving forward, we anticipate a rich interplay between experiments and theory for a broad range of solid materials. For example, experimental studies probing elastocapillary mechanics in micro-structured polymer gels and elastomers^28–32^ could inform our understanding and design of nanostructured metals and semiconductors^11, 12, 33^. To make meaningful comparisons between these very different systems, the relative size of the elastocapillary length and the length scale of deformation should be consistent.

Numerous applications already rely on compliant solids, from adhesives to soft robotics to medical implants^34–38^. Our results have immediate consequences for elastocapillary phenomena in these materials^32, 39–46^. Thanks to the strong strain dependence of the surface stress, the elastocapillary length scale can be tuned and significantly extended through careful control of the stress state. This suggests an exciting new design space where adhesion and wetting properties of a soft material could be modulated with mechanical stimuli. To fully realize these ideas, a fundamental investigation of the structure–property relationships that determine the surface modulus in polymeric materials is required. We expect that much can be learned from comparison to complex fluid-fluid interfaces, where a sophisticated understanding is emerging of the structure-property relationships that underlie surface rheology^47, 48^.

## Methods

### Silicone gel preparation and mechanical characterization

For the adhesion experiments, we prepare the silicone gel substrates by mixing liquid (1 Pa s^−1^) divinyl-terminated polydimethylsiloxane (PDMS) (Gelest, DMS-V31) with a chemical cross-linker (Gelest, HMS-301) and catalyst (Gelest, SIP6831.2), as described in detail in earlier work^21, 32^. We degas the silicone mixture in vacuum, form it into the desired experimental geometry, and cure the polymer at 68 °C for 12–14 h before the experiments. The resulting solid gel has a shear modulus of *G*′ = 1.9 kPa, as measured by bulk rheology, and the Poisson ratio of the gel’s elastic network is *ν* = 0.48, measured using a compression test in the rheometer as described in ref. ^49^. We also performed bulk tensile testing in order to measure the Young modulus as a function of strain. We find that the gel is linear elastic to ~10% true strain, then moderately strain stiffening thereafter (Supplementary Fig. 1). We observe no plastic deformation or flow in either shear rheology or in cyclic bulk tensile testing. For the wetting experiments, we prepare the silicone gel substrates from commercially available Dow Corning CY52-276. We mix the solutions of part A and part B with 1:1 volume ratio, and degas the mixture in vacuum. After waiting 45–50 min to let the mixture become slightly more viscous, we spin coat the mixture on top of a stiff silicone membrane (SMI/silicone sheet, Young’s Modulus around 1 MPa) at 800 r.p.m. for 1 min. As a result, a layer of uncured silicone with a thickness of ~ 80 μm is coated on top of the stiff membrane. Then we cure overnight at room temperature to produce a soft silicone gel. After curing, the Young modulus is *E* = 3.0 kPa and the Poisson ratio is 0.496. For detailed rheological data on this material, see ref. ^49^.

### Adhesion experiments

In the adhesion experiments, we directly image the deformation of the silicone gel during contact and subsequent quasi-static separation using an inverted optical microscope. We illuminate the sample with a low-Numerical Aperture (N.A.) condenser and image using a ×40 (N.A.= 0.60) air objective. To prepare the gel substrates in an appropriate geometry for visualization, we deposit a ~300-μm-thick layer of PDMS along the millimeter-wide edge of a standard microscope slide, as in the zero-force brightfield experiments of ref. ^21^. The silicone surface is flat parallel to the long edge of the microscope slide and very slightly curved (radius of curvature ~700 μm) in the orthogonal direction. This creates a very-nearly flat solid silicone surface that presents a flat edge clearly visible from the side. For the rigid, spherical indenters, we use untreated silica spheres (Polysciences, 07668) attached to the tapered ends of initially 1-mm-wide glass rods pulled to roughly 10 μm in diameter. We use two-part 5-min epoxy (Elmer’s) to attach the spheres to the rods, waiting 6–10 min after mixing before applying the glue to the spheres. This ensures that the glue does not flow over the sphere and change its surface properties. By mounting the glass rods on a three-axis micromanipulator stage (Narshige MMO-023), we are able to control manually the position of the individual spheres with sub-micrometer precision. Although high-speed imaging indicates that the initial contact deformation is complete in about a second, we allow the initial contact to equilibrate for ~10 min after first touch. We then quasi-statically move the spheres away from the surfaces (*D* < 0) by slowly moving the micromanipulator by 2 μm every 20 s, taking an image of the sphere adhered to the deformed substrate just before each step. We confirmed that these are stable configurations by extending the time between steps to 60 s, as well as by leaving a sphere and substrate in an adhered but highly deformed configuration for up to an hour, and observed no difference in the substrate deformation profile. We continue to increase the separation between sphere and silicone gel substrate until the adhesion becomes unstable. Given the small size of the particles, we would expect gravity to be negligible, but as the images are taken from the side, gravity is always normal to the plane of the images and hence has no role in the substrate deformation. We process each brightfield image from the adhesion experiments to locate the surface with precision of about 100 nm over a 427-μm field of view using the method and software developed in ref. ^21^ and described in detail therein. We measure the contact point between the adhered spheres and the PDMS surface, as well as the curvature of the surface near contact by fitting the measured profiles, with a shape that is the intersection of two surfaces of constant total curvature that meet at the contact line. We use the same algorithm and software developed for and described in detail in ref. ^21^, with the exception that we fix the contact angle to zero based on previous measurements^21^. From these fits, we measure the domain of constant curvature as the path length over which the constant curvature fit describes the data well, defined as the region over which the fit residuals remain small.

### Predictions of linear elastic theory

The theoretical surface profiles for the adhesion of a sphere to a linear-elastic half space (with no surface stress) are calculated using the theory of Maugis^18^. For each imaged profile, we measure *a* and *d*, and combine this with the known value of *R* to generate theoretical curves from Eq. (5) of ref. ^18^ without the need for any fitting parameters.

### Biaxial stretcher

It is based of the design of ref. ^50^ (Supplementary Fig. 2). It consists of two coaxial cylinders that are sealed together at the bottom. The membrane coated with soft silicone gel is placed flat on the top of the cylinders, and forms an upper seal for the air in the cavity between the two cylinders. This trapped air is then connected to an external syringe pump, with which we can control the cavity air pressure. Reducing this air pressure sucks the membrane into the cavity, creating a tension in the membrane and biaxially stretching the part over the inner cylinder. With this approach, we can achieve a maximum strain of ~25%.

### Macroscopic contact angle measurements

The macroscopic contact angles of glycerol droplets are measured by imaging back-lit droplets on soft substrates from the side with a CMOS camera (DCC3240C, Thorlabs). We observed that for all substrates both advancing and receding droplets always equilibrated to the same contact angle, so we find no contact angle hysteresis (Supplementary Fig. 3).

### Confocal imaging of the wetting profile

We imaged the microscopic profile of the soft substrates under the contact line of glycerol droplets by tracking the position of small fluorescent nanobeads attached to the soft silicone substrate with a confocal microscope. This used the same procedure as that in refs. ^16, 26^, with the only difference being that we used 48-nm-diameter yellow/green fluorescent beads (Life Technologies, F-8795). The total areal coverage of beads is very small: we estimate it as being 0.4% of the surface area. Thus, we do not expect the presence of beads to significantly affect the surface properties of the silicone.

### Local strain measurements

In order to measure the local strain near the contact point, we measure the local in-plane and out-of-plane displacements of the substrate, (*u*_*x*_, *u*_*z*_), near the contact line by tracking the displacements of individual fluorescent beads upon removing a glycerol droplet (e.g., see ref. ^26^ for details). An example is shown in Supplementary Fig. 6.

We then convert these displacements into the extra strain due to the presence of the wetting ridge, Δ$\epsilon$, using the relation:

2 $$\Delta \epsilon = \sqrt {{{\left( {1 + \frac{{\partial {u_x}}}{{\partial x}}} \right)}^2} + {{\left( {\frac{{\partial {u_z}}}{{\partial x}}} \right)}^2}} - 1$$

The local strain at the wetting ridge is then $\epsilon$ = ${\epsilon _\infty }$ + Δ$\epsilon$.

### Measurement of liquid–air surface tension

The surface tension of glycerol droplets on silicone gel substrates was measured by analyzing the shape of large sessile droplets on flat gel surfaces. Droplets, with spread diameters of ~10 mm, were placed on a substrate and allowed to equilibrate for at least an hour. The droplets were then imaged from the side, and we used a bespoke Matlab program to extract their shape. Finally, we obtained surface tension values by fitting these measured shapes with theoretical profiles generated from the Bashforth–Adams equation, following ref. ^51^. The surface tension of uncured silicone was measured using the pendant droplet method. We hung the silicone-liquid droplet from the end of a blunt-tipped needle, and imaged it from the side. Again, we extracted the shape of the droplet using a Matlab program and obtained the surface tension by fitting this shape with the Bashforth–Adams equation^51^.

### Data availability

The data that support the findings of this study are available from the authors on request.

## Electronic supplementary material

Supplementary Information

## References

[1] Shuttleworth R (1950). The surface tension of solids. Proc. Phys. Soc. A.

[2] Gurtin ME, Murdoch AI (1975). A continuum theory of elastic material surfaces. Arch. Rational Mech. Anal.

[3] Spaepen F (2000). Interfaces and stresses in thin films. Acta Mater..

[4] Vanderbilt D (1987). Absence of large compressive stress on Si (111). Phys. Rev. Lett..

[5] Gumbsch P, Daw MS (1991). Interface stresses and their effects on the elastic moduli of metallic multilayers. Phys. Rev. B.

[6] Shenoy VB (2005). Atomistic calculations of elastic properties of metallic fcc crystal surfaces. Phys. Rev. B.

[7] Miller RE, Shenoy VB (2000). Size-dependent elastic properties of nanosized structural elements. Nanotechnology.

[8] Dingreville R, Qu J, Cherkaoui M (2005). Surface free energy and its effect on the elastic behavior of nano-sized particles, wires and films. J. Mech. Phys. Solids.

[9] Duan HL, Wang J, Huang ZP, Karihaloo BL (2005). Eshelby formalism for nano-inhomogeneities. Proc. R. Soc. A.

[10] Sharma P, Ganti S (2004). Size-dependent eshelby’s tensor for embedded nano-inclusions incorporating surface/interface energies. J. Appl. Mech..

[11] He J, Lilley CM (2008). Surface effect on the elastic behavior of static bending nanowires. Nano Lett..

[12] Lu D, Xie YM, Li Q, Huang X, Zhou S (2014). Towards ultra-stiff materials: surface effects on nanoporous materials. Appl. Phys. Lett..

[13] Diehm PM, Ágoston P, Albe K (2012). Size-dependent lattice expansion in nanoparticles: reality or anomaly?. Chemphyschem.

[14] Wolfer WG (2011). Elastic properties of surfaces on nanoparticles. Acta Mater..

[15] Cammarata R, Sieradzki K (1994). Surface and interface stresses. Ann. Rev. Mater. Sci.

[16] Style RW, Che Y, Wettlaufer JS, Wilen LA, Dufresne ER (2013). Universal deformation of soft substrates near a contact line and the direct measurement of solid surface stresses. Phys. Rev. Lett..

[17] Johnson K, Kendall K, Roberts A (1971). Surface energy and the contact of elastic solids. Proc. R. Soc. A.

[18] Maugis D (1995). Extension of the Johnson-Kendall-Roberts theory of the elastic contact of spheres to large contact radii. Langmuir.

[19] Liu T, Jagota A, Hui C.-Y (2016). Effect of surface tension on the adhesion between a rigid flat punch and a semi-infinite neo-Hookean half-space. Extreme Mech. Lett..

[20] Style RW, Jagota A, Hui C-Y, Dufresne ER (2016). Elastocapillarity: surface tension and the mechanics of soft solids. Annu. Rev. Condens. Matter Phys..

[21] Jensen KE (2015). Wetting and phase separation in soft adhesion. Proc. Natl Acad. Sci. USA.

[22] Style RW, Dufresne ER (2012). Static wetting on deformable substrates, from liquids to soft solids. Soft Matter.

[23] Park SJ (2014). Visualization of asymmetric wetting ridges on soft solids with x-ray microscopy. Nat. Commun..

[24] Bostwick JB, Shearer M, Daniels KE (2014). Elastocapillary deformations on partially-wetting substrates: rival contact-line models. Soft Matter.

[25] Cao Z, Dobrynin AV (2015). Polymeric droplets on soft surfaces: from Neumann’s triangle to Young’s law. Macromolecules.

[26] Jerison ER, Xu Y, Wilen LA, Dufresne ER (2011). Deformation of an elastic substrate by a three-phase contact line. Phys. Rev. Lett..

[27] Ibach H (1997). The role of surface stress in reconstruction, epitaxial growth and stabilization of mesoscopic structures. Surf. Sci. Rep..

[28] Jagota A, Paretkar D, Ghatak A (2012). Surface-tension-induced flattening of a nearly plane elastic solid. Phys. Rev. E.

[29] Nadermann N, Hui C-Y, Jagota A (2013). Solid surface tension measured by a liquid drop under a solid film. Proc. Natl Acad. Sci. USA.

[30] Mora S, Phou T, Fromental J-M, Pismen LM, Pomeau Y (2010). Capillarity driven instability of a soft solid. Phys. Rev. Lett..

[31] Ducloue L, Pitois O, Goyon J, Chateau X, Ovarlez G (2014). Coupling of elasticity to capillarity in soft aerated materials. Soft Matter.

[32] Style RW (2015). Stiffening solids with liquid inclusions. Nat. Phys.

[33] Chen CQ, Shi Y, Zhang YS, Zhu J, Yan YJ (2006). Size dependence of youngs modulus in zno nanowires. Phys. Rev. Lett..

[34] Creton C, Papon E (2003). Materials science of adhesives: how to bond things together. MRS Bull..

[35] Kim S, Laschi C, Trimmer B (2013). Soft robotics: a bioinspired evolution in robotics. Trends Biotechnol..

[36] Minev IR (2015). Electronic dura mater for long-term multimodal neural interfaces. Science.

[37] Drury JL, Mooney DJ (2003). Hydrogels for tissue engineering: scaffold design variables and applications. Biomaterials.

[38] Rose S (2014). Nanoparticle solutions as adhesives for gels and biological tissues. Nature.

[39] Andreotti B (2016). Solid capillarity: when and how does surface tension deform soft solids?. Soft Matter.

[40] Andreotti B, Snoeijer JH (2016). Soft wetting and the shuttleworth effect, at the crossroads between thermodynamics and mechanics. Europhys. Lett..

[41] Chakrabarti A, Chaudhury MK (2013). Direct measurement of the surface tension of a soft elastic hydrogel: Exploration of elastocapillary instability in adhesion. Langmuir.

[42] Style RW, Hyland C, Boltyanskiy R, Wettlaufer JS, Dufresne ER (2013). Surface tension and contact with soft elastic solids. Nat. Commun.

[43] Style RW (2013). Patterning droplets with durotaxis. Proc. Natl Acad. Sci. USA.

[44] Gonzalez-Rodriguez D (2015). Elastocapillary instability in mitochondrial fission. Phys. Rev. Lett..

[45] Karpitschka S (2016). Liquid drops attract or repel by the inverted cheerios effect. Proc. Natl Acad. Sci. USA.

[46] Jensen, K. E., Style, W. R., Xu, Q. & Dufresne, R. E. Strain-dependent solid surface stress and the stiffness of soft contacts. Preprint at http://arxiv.org/abs/1707.03089 (2017).

[47] Fuller GG, Vermant J (2012). Complex fluid-fluid interfaces: rheology and structure. Ann. Rev. Chem. Biomol. Eng..

[48] Hermans E, Bhamla MS, Kao P, Fuller GG, Vermant J (2015). Lung surfactants and different contributions to thin film stability. Soft Matter.

[49] Style RW (2014). Traction force microscopy in physics and biology. Soft Matter.

[50] Na S (2008). Time-dependent changes in smooth muscle cell stiffness and focal adhesion area in response to cyclic equibiaxial stretch. Ann. Biomed. Eng..

[51] del Rio O, Neumann AW (1997). Axisymmetric drop shape analysis: computational methods for the measurement of interfacial properties from the shape and dimensions of pendant and sessile drops. J. Colloid. Interface Sci..

[52] Xu X, Jagota A, Paretkar D, Hui C-Y (2016). Surface tension measurement from the indentation of clamped thin films. Soft Matter.

[53] Mondal S, Phukan M, Ghatak A (2015). Estimation of solid liquid interfacial tension using curved surface of a soft solid. Proc. Natl Acad. Sci. USA.

