# Supplementary material for: Direct measurement of strain-dependent solid surface stress
Source: Nat Commun. 2017 Sep 15;8:555. doi: 10.1038/s41467-017-00636-y (PMC5601460; doi:10.1038/s41467-017-00636-y)
Supplement: Supplementary file 1 — Supplementary Information [file 41467_2017_636_MOESM1_ESM.pdf]

Description of Supplementary Files

File Name: Supplementary Information
Description: Supplementary Figures

Supplemental Figures

July 13, 2017

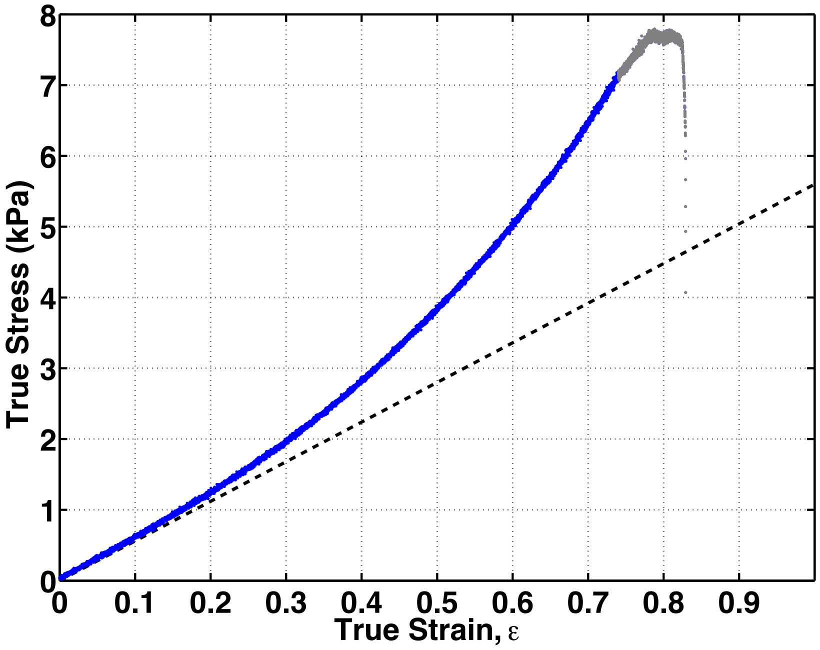

Figure 1: **Tensile test.** True stress vs. true strain for a bulk tensile test of the Gelest silicone used for the adhesion experiments. Dashed line has a 5.6 kPa slope, corresponding to the low-strain elastic modulus. In this experiment, the sample failed by tearing from the tensile grips beginning at 74% true strain.

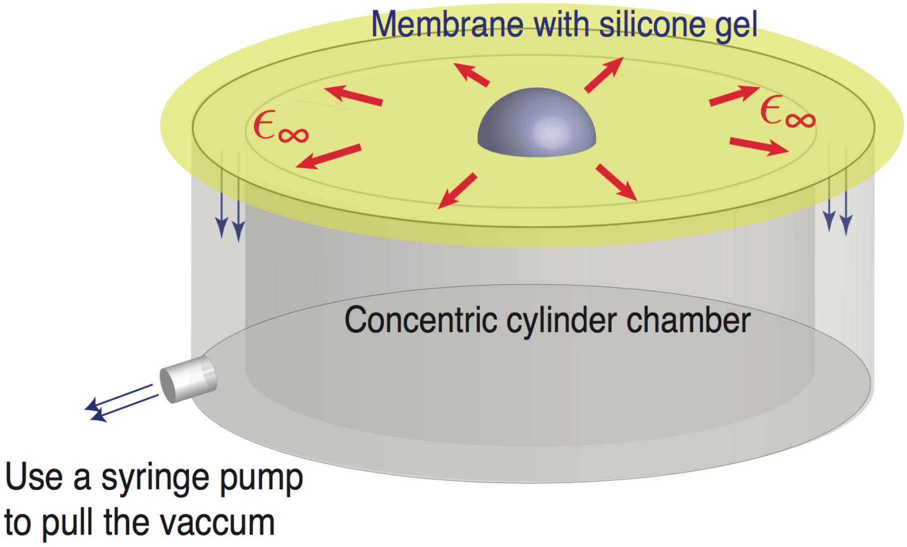

Figure 2: **Schematic illustration of the bi-axial stretcher.** The part of the membrane over the inner cylinder is where we perform our experimental observations. Droplets and fluorescent beads attached to this region of the membrane are imaged from below using an inverted confocal microscope. The precise bi-axial strain, ϵ_∞ exerted on the membrane is calculated by tracking fluorescent beads attached to the membrane

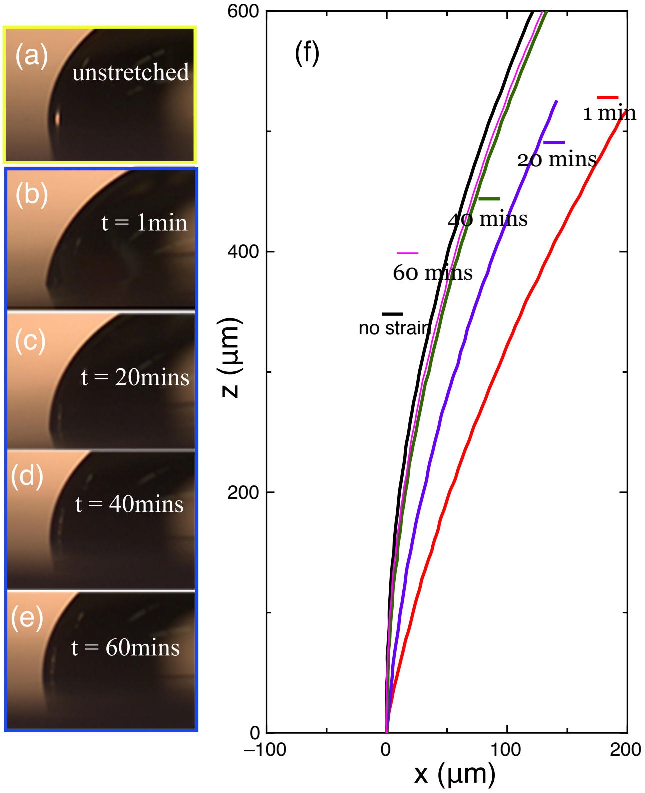

Figure 3: **Macroscopic contact angle measurements.** (a) The glycerol droplet profile on an a unstretched soft gel surface. (b)-(e) The relaxation of droplet profiles after a sudden stretch with 10% strain (after 1min, 20mins, 40mins, 60mins, respectively). (f) Measured drop profiles obtained from the image analysis. The droplet fully relax to a constant contact angle of $\theta = 90.8 \pm 0.2^o$, which is identical to the contact angle found for the unstretched droplet.

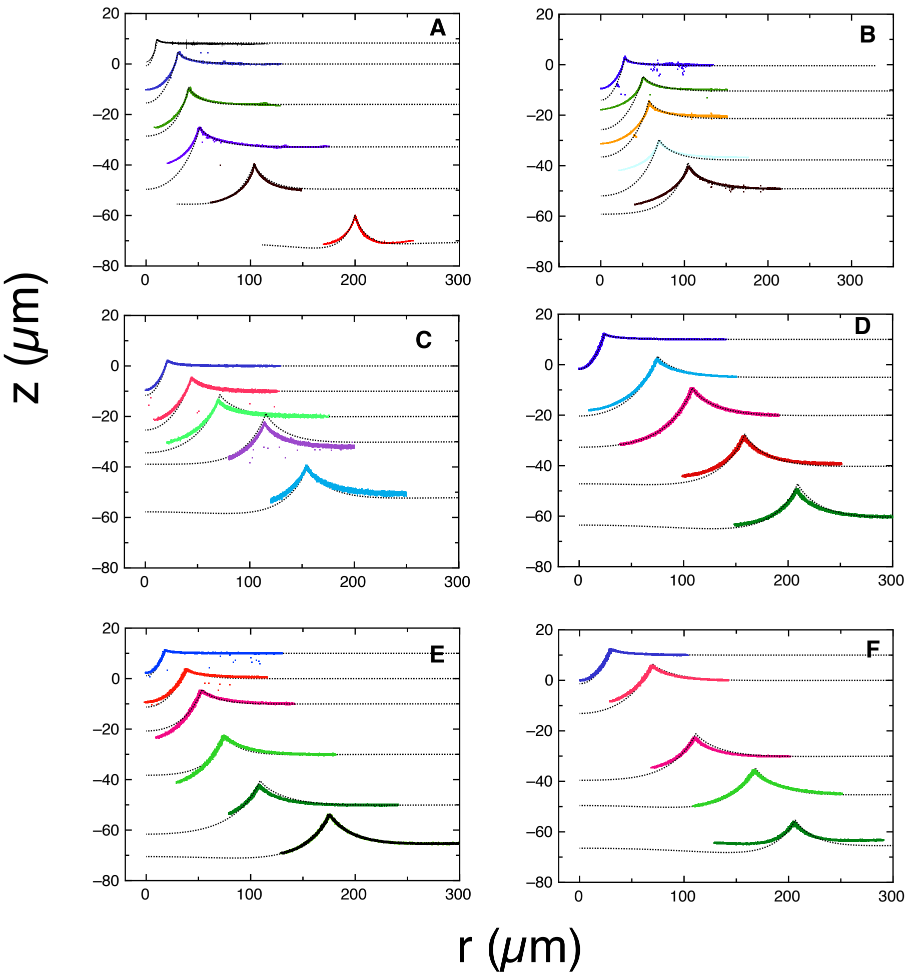

Figure 4: **Wetting profiles at different applied strains, ϵ_∞. (A)-(F)**: $\epsilon_\infty = 0, \epsilon_\infty = 0.07, \epsilon_\infty = 0.09, \epsilon_\infty = 0.014, \epsilon_\infty = 0.18, \epsilon_\infty = 0.23$, respectively. The black dashed curves are theoretical calculations using the model from Ref. [22] and the measured values of $\Upsilon(\epsilon)$ using the measured values of $\Upsilon_g(\epsilon)$ obtained in our experiments. . In these calculations, the substrate thicknesses at different applied strains are estimated as $h = h_0(1 - \epsilon_\infty)^2$ where h_0 is the unstretched thickness. This follows as Poisson's ratio for our silicone gel is very nearly 0.5.

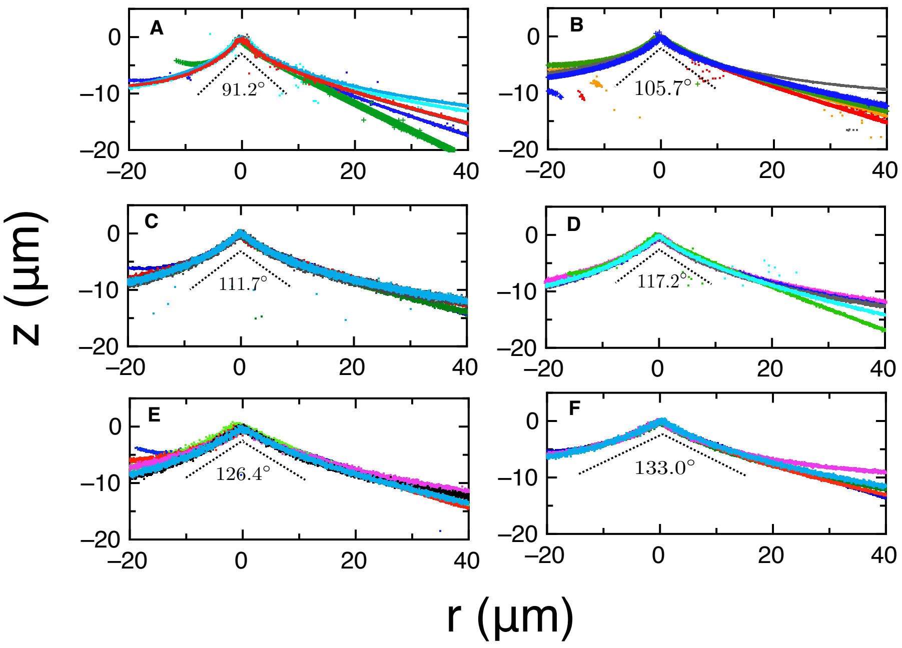

Figure 5: **Collapse of droplets near the contact point.** Each of the drop profiles in Fig.S4 are translated and rotated to collapse the plots near the contact point. **(A)-(F)**: $\epsilon_\infty = 0, \epsilon_\infty = 0.07, \epsilon_\infty = 0.09, \epsilon_\infty = 0.014, \epsilon_\infty = 0.18, \epsilon_\infty = 0.23$.

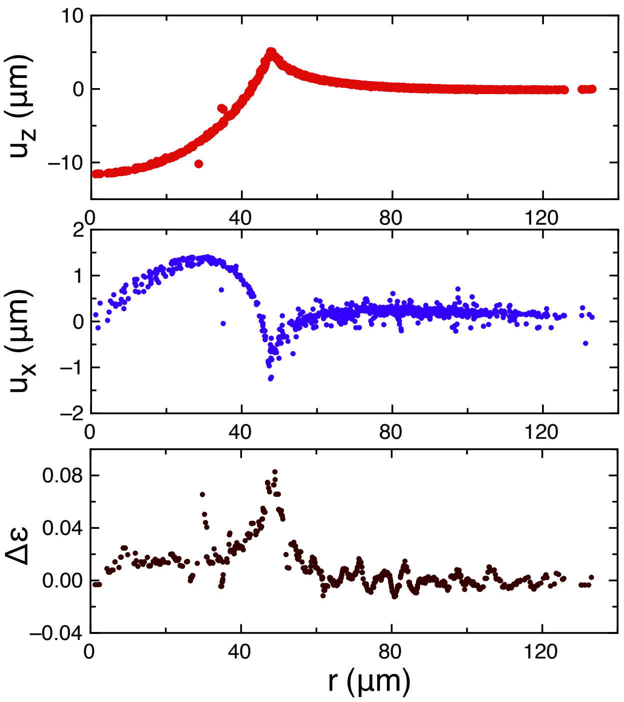

Figure 6: Example of the local strain calculations.
